# Trends in Urgent Care Utilization Among Medicare Beneficiaries From 2012 to 2019

**DOI:** 10.1001/jamanetworkopen.2025.55345

**Published:** 2026-01-26

**Authors:** Joel J. Mantilla, Ryan C. Burke, E. John Orav, Barbara A. Masser, Richard E. Wolfe, Amber K. Sabbatini, Michelle P. Lin, Ari B. Friedman, Laura G. Burke

**Affiliations:** 1 Herbert Wertheim College of Medicine, Florida International University, Miami; 2 Department of Emergency Medicine, Brown University Health Medical Group, Providence, Rhode Island; 3 Department of Health Policy and Management, Harvard T.H. Chan School of Public Health, Boston, Massachusetts; 4 Department of Medicine, Division of General Internal Medicine, Brigham and Women’s Hospital, Boston, Massachusetts; 5 Department of Emergency Medicine, Beth Israel Deaconess Medical Center, Boston, Massachusetts; 6 Department of Emergency Medicine, University of Washington, Seattle; 7 Department of Emergency Medicine, Stanford University, Palo Alto, California; 8 Department of Emergency Medicine, University of Pennsylvania, Philadelphia

## Abstract

**Importance:**

Urgent care (UC) centers have proliferated rapidly, yet research on how utilization has changed among older adults is limited.

**Objectives:**

To examine UC utilization among older adults and assess whether utilization rates varied by beneficiary sociodemographic and community characteristics.

**Design, Setting, and Participants:**

This cross-sectional study used data from a 20% national sample of fee-for-service Medicare beneficiaries aged 65 years or older using UC centers from January 1, 2012, to December 31, 2019. Statistical analysis was performed from May 1, 2021, to November 24, 2025.

**Main Outcome and Measures:**

Among Medicare beneficiaries aged 65 years or older, unadjusted UC visits were calculated by year from 2012 to 2019 overall and stratified by demographic characteristics, frailty, community rurality, Social Deprivation Index (SDI), and physician supply. Adjusted incidence rate ratios (IRRs) were calculated for UC visits in 2018 and 2019 using negative binomial models. Trends in the distribution of UC visits among the most frequent clinician specialty categories (primary care, emergency medicine, and advanced practice practitioners [APPs]) were examined using linear models.

**Results:**

There were 3 516 816 UC visits among 9 514 946 beneficiaries (mean [SD] age across visits, 75.2 [7.5] years; 63.4% women). UC visits increased from 47.7 to 117.2 per 1000 from 2012 to 2019 (9.0 [95% CI, 9.0-9.1] visits per 1000 per year). The growth in UC utilization was slowest for beneficiaries aged 85 years or older (4.0 [95% CI, 3.8-4.1] visits per 1000 per year), Medicaid-eligible beneficiaries (4.0 [95% CI, 3.9-4.2] visits per 1000 per year), those residing in communities that were rural (5.0 [95% CI, 4.8-5.2] visits per 1000 per year), thow who were disadvantaged (6.8 [95% CI, 6.0-7.6] visits per 1000 per year), and those with fewer physicians (7.2 [95% CI, 5.5-8.8] visits per 1000 per year). In 2018 and 2019, beneficiaries residing in rural communities had 45% lower adjusted UC utilization compared with urban communities (IRR, 0.55 [95% CI, 0.54-0.55]) and those residing in zip codes in the SDI fourth quartile had 23% lower adjusted UC utilization (IRR, 0.77 [95% CI, 0.77-0.78]) compared with those in the most advantaged quartile. The percentage of beneficiaries managed by APPs increased from 21.0% in 2012 to 50.8% in 2019.

**Conclusions and Relevance:**

In this cross-sectional analysis, UC utilization increased markedly among older adults, with a disproportionate concentration in urban, less-disadvantaged communities. The distribution of clinician training and specialty also changed, with APPs delivering care for more than half of UC visits among older adults in 2019. These findings highlight the evolving patterns of acute care delivery for this growing population and the need for additional evidence on how these trends are associated with patient-centered outcomes and the efficiency of care.

## Key Points

**Question:**

How has urgent care (UC) utilization changed among older adults, and to what degree has utilization varied by patient and community characteristics?

**Findings:**

In this cross-sectional study, UC visits among older adults more than doubled from 2012 to 2019, with the slowest growth among beneficiaries aged 85 years or older, eligible for Medicaid, and residing in rural and disadvantaged communities. There was a marked increase in UC visits to advanced practice practitioners (APPs), who managed more than half of visits in 2019.

**Meaning:**

This study suggests that older adults have increasingly used UC for acute, unscheduled care, although access appears to be limited for vulnerable populations.

## Teaser

This cross-sectional study examines urgent care utilization among adults aged 65 years or older and assesses whether utilization rates varied by beneficiary sociodemographic and community characteristics.

## Introduction

Urgent care (UC) centers have proliferated rapidly in response to the perceived need for acute, unscheduled care.^1,2,3^ Payers and policymakers have promoted UC as a cost-effective alternative to emergency departments (EDs), particularly for low-acuity medical conditions, despite evidence that it may increase net health care spending overall.^2,4^ Older adults, particularly those who are Medicaid eligible, members of racial and ethnic minority groups, and those with more chronic diseases, have higher utilization rates of acute unscheduled care,^5^ yet their use of UC services remains poorly characterized. Most studies of UC have focused on younger populations and the commercially insured.^3,6,7,8^ How UC utilization has changed among older adults and the degree to which patient (eg, frailty, socioeconomic status) or community characteristics (eg, physician supply, urbanicity) are associated with UC utilization among older adults has received little attention.

Furthermore, UC differs from other ambulatory settings in that the clinical capabilities—including clinician training and specialty—can vary widely.^6^ Physicians trained in family medicine, internal medicine, and emergency medicine, as well as advanced practice practitioners (APPs; eg, nurse practitioners and physician assistants) all commonly practice in UC settings.^6^ However, whether the distribution of clinicians has changed with increasing UC availability is unknown.

We examined national claims for Medicare beneficiaries aged 65 years or older from 2012 to 2019 to address the following questions. First, how has UC utilization changed among older adults? Second, have rates of UC visits and trends over time varied by beneficiary clinical, sociodemographic, and community characteristics? Third, which types of clinicians provide UC services to older adults, and has this changed over time?

## Methods

### Study Sample, Data Sources, and Outcomes

This cross-sectional study included UC visits in the US among a 20% sample of beneficiaries of traditional, fee-for-service Medicare, aged 65 years or older, from January 1, 2012, to December 31, 2019. We excluded visits during the COVID-19 pandemic, given the large disruptions in ambulatory care delivery,^9,10^ including unscheduled care.^11,12,13^ The Office of Human Research Administration at the Harvard School of Public Health approved this study. Because this was a retrospective study of previously collected data, obtaining informed consent was not feasible. The study was conducted in accordance with the Strengthening the Reporting of Observational Studies in Epidemiology (STROBE) reporting guideline for reporting observational research.

UC visits were identified from Medicare carrier professional claims using evaluation and management Healthcare Common Procedure Coding System or *Current Procedural Terminology* codes (99201-99205 and 99211-99215) with a place of service code of UC.^2,14,15^ Beneficiary and community characteristics were defined yearly using beneficiary zip codes and assigned to each visit (eAppendix in Supplement 1). We identified the clinician specialty on each professional claim and the following beneficiary characteristics from the Master Beneficiary Summary File: age, sex, Medicaid eligibility, and race and ethnicity (American Indian or Alaska Native, Asian or Pacific Islander, Black, Hispanic, White, other race or ethnicity [all beneficiaries who could not be assigned to 1 of the other 5 categories of race and ethnicity], and unknown race or ethnicity). Race and ethnicity were examined to assess disparities in UC access using the Research Triangle Institute Variable, which is derived from administrative sources and not self-report.^16^ Hispanic ethnicity is listed as a separate category that cannot be combined with race and ethnicity categories.

We identified 26 beneficiary chronic conditions from the Chronic Conditions Warehouse file. Given the independent association of frailty among older adults with health care outcomes, utilization, and spending,^17,18^ a frailty score was calculated using established methods.^19,20^ Given evidence suggesting that UC centers have preferentially entered wealthy, urban communities,^21^ we examined if these community characteristics are associated with differential trends in UC utilization among older adults. We linked beneficiaries’ 9-digit zip codes with Rural-Urban Commuting Area (RUCA) codes, defining a RUCA of 4 or more as rural and all others as urban. We created quartiles of beneficiary zip codes with respect to the Social Deprivation Index^22^ (SDI; years 2015-2019) as an indicator of community-level socioeconomic status. In addition, we examined if UC utilization varied by area-level physician supply, as UC entry could address unmet demand due to clinician shortages. To do so, we assigned each beneficiary to a Dartmouth Atlas Hospital Referral Region (HRR). HRRs represent regional markets for tertiary medical care used to study health care organization, delivery, and outcomes.^23^ We created quartiles of HRRs with respect to physicians per 100 000 population.

### Statistical Analysis

#### Trends in UC Utilization Overall and by Beneficiary and Community Characteristics

Data were analyzed from May 1, 2021, to November 24, 2025. We first plotted annual UC visits per 1000 eligible beneficiaries overall and stratified by patient and community characteristics. We tested for time trends in these univariate associations using a beneficiary year–level linear regression model with UC visits during the year as the outcome and year as the linear independent variable, and incorporating beneficiary fixed effects to adjust for correlation over time. We did this for all beneficiaries, as well as separately for each patient and community characteristic as categorical variables, with an interaction between year and the respective characteristic to identify differential trends.

#### Variation in UC Utilization by Patient and Community Characteristics

To examine if key patient and community characteristics were associated with UC utilization in the immediate prepandemic period, we first calculated unadjusted utilization rates per 1000 eligible beneficiary years in 2018 and 2019. We then calculated adjusted incidence rate ratios (IRRs) using a negative binomial regression model, with UC visits per population as the outcome and beneficiary and community characteristics (eAppendix in Supplement 1) as variables, adjusting for beneficiary chronic conditions as covariates.

#### Trends in Clinician Specialty Providing UC Services

We categorized clinician specialty for each visit and summarized the absolute number of visits as well as the proportion of visits attributed to each specialty. We then examined time trends in UC utilization for the 6 most common specialties. We also examined the proportion of visits attributed to each specialty using linear regression models with year and specialty as the variables, as well as an interaction between year and specialty, and beneficiary fixed effects to account for correlation over multiple years of data.

We analyzed data using SAS, version 9.4 (SAS Institute Inc). Results were considered significant at a 2-sided *P* < .05.

## Results

### Study Sample Characteristics

Our sample (Table 1) included 3 516 816 UC visits among 9 514 946 unique Medicare beneficiaries (mean [SD] age across visits, 75.2 [7.5] years; 63.4% women and 36.6% men; 0.3% American Indian or Alaska Native, 1.7% Asian or Pacific Islander, 4.3% Black, 4.0% Hispanic, 87.6% White, 0.7% other race or ethnicity, and 1.4% unknown race or ethnicity). Of all beneficiaries in the sample, 15.2% had at least 1 UC visit, with a mean (SD) of 0.4 (1.5) visits per beneficiary. The mean (SD) beneficiary age for UC visits was 75.4 (7.6) years in 2012 and 75.1 (7.4) years in 2019. The number of visits among adults aged 85 years or older was 37 545 of 252 926 (14.9%) in 2012 and 81 315 of 638 490 (12.7%) in 2019. The mean (SD) frailty score was 0.17 (0.07) in 2012 vs 0.16 (0.06) in 2019. The percentage of visits among female beneficiaries was similar in 2012 vs 2019 (Table 1).

**Table 1.** Sample Characteristics of Urgent Care Visits Among Medicare Beneficiaries, 2012-2019^a^

Characteristic	No. (%)	
	2012 (252 926 Visits)	2019 (638 490 Visits)
Age, mean (SD), y	75.4 (7.6)	75.1 (7.4)
Frailty score, mean (SD)	0.17 (0.07)	0.16 (0.06)
Age category, y		
65-74	131 781 (52.1)	348 533 (54.6)
75-84	83 600 (33.1)	208 642 (32.7)
≥85	37 545 (14.8)	81 315 (12.7)
Sex		
Male	93 523 (37.0)	234 679 (36.8)
Female	159 403 (63.0)	403 811 (63.2)
Race and ethnicity^b^		
American Indian or Alaska Native	826 (0.3)	1660 (0.3)
Asian or Pacific Islander	3348 (1.3)	12 822 (2.0)
Black	9959 (3.9)	27 989 (4.4)
Hispanic	9945 (3.9)	26 171 (4.1)
White	226 599 (89.6)	552 467 (86.5)
Other	1360 (0.5)	4492 (0.7)
Unknown	889 (0.4)	12 889 (2.0)
Medicaid eligibility		
Not Medicaid eligible	233 619 (92.4)	593 698 (93.0)
Medicaid eligible	19 307 (7.6)	44 792 (7.0)
Condition prevalence		
Alzheimer disease and related dementias	16 354 (6.7)	42 020 (6.7)
Congestive heart failure	30 762 (12.6)	72 362 (11.6)
Depression	33 426 (13.6)	112 815 (18.1)
Diabetes	60 099 (24.5)	152 940 (24.5)
Ischemic heart disease	77 640 (31.7)	176 686 (28.3)

^a^ Random 20% sample of fee-for-service Medicare beneficiaries aged 65 years or older in the 50 US states and the District of Columbia.

^b^ Defined using the Research Triangle Institute Variable. “Other” refers to all beneficiaries who cannot be assigned to 1 of the other 5 categories of race and ethnicity, whereas “Unknown” refers to individuals lacking any administrative data on race and ethnicity.

### Trends in UC Utilization

**Figure 1.**
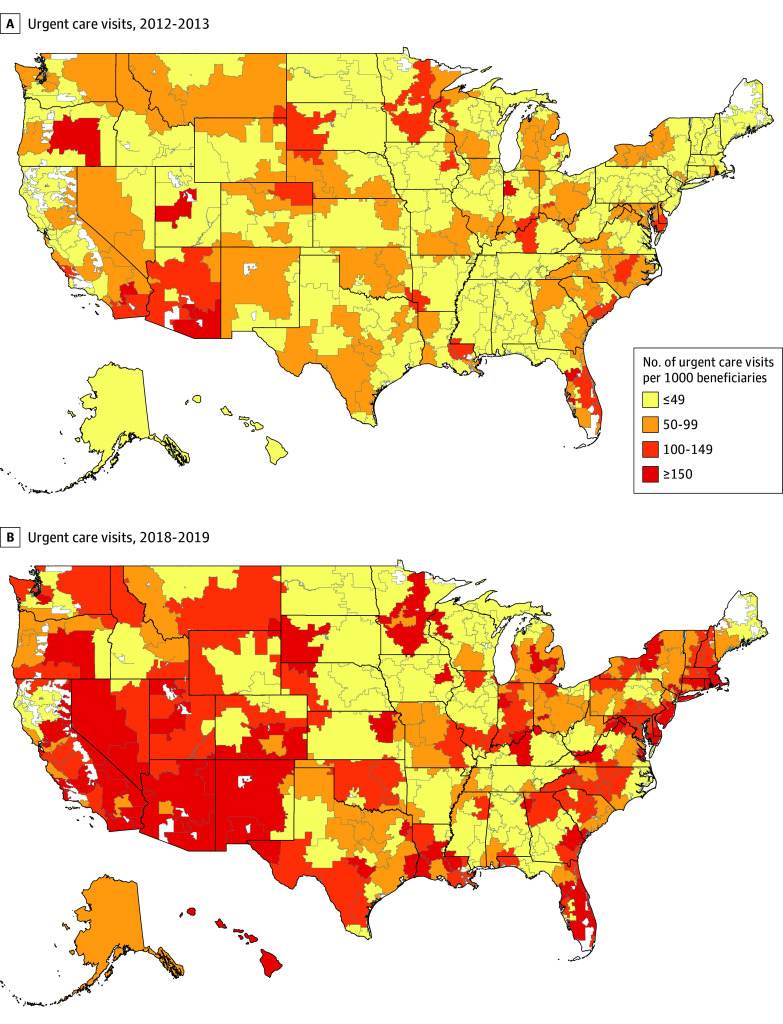
Trends in Urgent Care Utilization in 2012 and 2013 vs 2018 and 2019 Among Medicare Beneficiaries Hospital Referral Regions (HRRs) were obtained from the Dartmouth Atlas and represent regional markets for tertiary medical care used to study health care organization, delivery, and outcomes. HRRs were determined yearly and assigned based on beneficiary zip code. Twenty percent of the national sample of Medicare beneficiares aged 65 years or older enrolled in Parts A and B.

UC utilization increased by 9.0 (95% CI, 9.0-9.1) visits per 1000 per year, from 47.7 UC visits per 1000 beneficiaries in 2012 to 117.2 visits per 1000 in 2019 (Table 2). These trends varied by community and beneficiary characteristics (Table 2 and Figure 1; eFigure 1 in Supplement 1). Although all age groups showed an increase, this growth was greatest for beneficiaries aged 65 to 74 years (11.0 [95% CI, 10.7-11.4] visits per 1000 per year) and the least among those aged 85 years or older (4.0 [95% CI, 3.8-4.1] visits per 1000 per year). The association between frailty and trends in UC utilization was not monotonic; beneficiaries in the third frailty quartile had the greatest increase (10.1 [95% CI, 9.6-10.6] visits per 1000 per year), and those in the fourth quartile (ie, the most frail) had the smallest increase (5.9 [95% CI, 5.4-6.4] visits per 1000 per year). Medicaid-eligible beneficiaries had lower baseline rates of UC visits in 2012 compared with those who were not Medicaid eligible (25.1 vs 51.5 visits per 1000) and lesser increases over time (4.0 [95% CI, 3.9-4.2] vs 9.8 [95% CI, 9.4-10.1] visits per 1000 per year). Beneficiaries with race and ethnicity listed as unknown or White showed greater increases in UC visit rates compared with all other categories.

**Table 2.** Trends in Urgent Care Visits Per 1000 Traditional Medicare Beneficiaries Aged 65 Years or Older, 2012-2019

Characteristic	No. of visits per 1000 beneficiaries^a^		Change in No. of visits per 1000 beneficiaries per year (95% CI)^b^
	2012	2019	
All beneficiaries	47.7	117.2	9.0 (9.0-9.1)
Age category, y			
65-74	48.8	118.6	11.0 (10.7-11.4)
75-84	48.2	120.4	8.5 (8.1-8.8)
≥85	43.1	105.0	4.0 (3.8-4.1)
Frailty index^c^			
First quartile (least frail)	36.4	86.2	8.5 (8.0-8.9)
Second quartile	49.2	118.0	9.8 (9.4-10.3)
Third quartile	54.3	136.1	10.1 (9.6-10.6)
Fourth quartile (most frail)	51.1	130.3	5.9 (5.4-6.4)
Missing	46.3	116.4	7.9 (7.7-8.1)
Sex			
Male	40.9	97.5	7.9 (7.9-8.0)
Female	52.8	132.9	9.8 (9.6-10.0)
Race and ethnicity^d^			
American Indian or Alaska Native	37.6	64.9	3.3 (2.5-4.1)
Asian or Pacific Islander	26.0	83.3	7.6 (6.0-9.3)
Black	24.8	73.0	5.7 (4.1-7.4)
Hispanic	35.4	92.6	7.6 (5.9-9.2)
White	51.4	124.1	9.4 (7.8-11.0)
Other	35.3	100.1	8.9 (7.1-10.7)
Unknown	42.1	123.2	13.2 (11.5-15.0)
Medicaid or eligibility			
Not Medicaid eligible	51.5	123.9	9.8 (9.4-10.1)
Medicaid eligible	25.1	68.3	4.0 (3.9-4.2)
Urban vs rural residence^e^			
Rural	29.1	68.3	5.0 (4.8-5.2)
Urban	53.5	132.4	10.3 (10.2-10.3)
Social Deprivation Index^f^			
First quartile (least disadvantaged)	58.8	145.6	11.9 (11.1-12.6)
Second quartile	52.5	121.0	9.1 (8.3-9.9)
Third quartile	41.4	99.0	7.3 (6.6-8.1)
Fourth quartile (most disadvantaged)	34.3	89.3	6.8 (6.0-7.6)
Missing	47.3	120.5	8.6 (8.3-9.0)
No. of physicians per 100 000 population^g^			
First quartile (fewest physicians)	43.9	101.2	7.2 (5.5-8.8)
Second quartile	52.1	110.9	7.6 (6.0-9.2)
Third quartile	60.5	123.5	7.6 (6.0-9.3)
Fourth quartile (most physicians)	35.5	134.0	13.5 (11.9-15.2)
Missing	16.9	32.5	1.7 (0.9-2.5)

^a^ Raw number of urgent care visits per 1000 traditional Medicare beneficiaries in the respective year.

^b^ Trends over time in urgent care visits per beneficiary as the outcome and year as the variable. Time trends for each beneficiary characteristic were obtained using separate linear regression models with each characteristic as a categorical variable and an interaction between year and the respective characteristic. The *P* values were less than .001 for each interaction between the characteristics shown above and year.

^c^ Frailty score was determined according to prior published claims-based methods.^20^

^d^ Race and ethnicity were defined using the Research Triangle Institute Variable. “Other” refers to all beneficiaries who cannot be assigned to 1 of the other 5 categories of race and ethnicity, whereas “Unknown” refers to individuals lacking any administrative data on race and ethnicity.

^e^ Beneficiaries residing in zip codes with a Rural-Urban Community Area code of 4 or greater were classified as having a rural residence and all others as urban.

^f^ Quartiles of community Social Deprivation Index based on beneficiary zip code, as an indicator of community-level socioeconomic status.

^g^ Each beneficiary was assigned to a Dartmouth Atlas Hospital Referral Region according to zip code. Physician supply for each Hospital Referral Region was obtained for the Dartmouth Atlas and quartiles of Hospital Referral Region–level physician supply were created.

Beneficiaries residing in zip codes in the first quartile of SDI (ie, least disadvantaged) had the greatest increase in UC visits (11.9 [95% CI, 11.1-12.6] visits per 1000 per year), while those in the fourth (most disadvantaged) quartile had the smallest increase over time (6.8 [95% CI, 6.0-7.6] visits per 1000 per year) (Table 2). Urban communities had more than double the increase in UC utilization compared with rural communities (10.3 [95% CI, 10.2-10.3] vs 5.0 [95% CI, 4.8-5.2] visits per 1000 per year), and there was a monotonic positive association between HRR-level physician supply and trends in UC visits (fewest physicians: 7.2 [95% CI, 5.5-8.8] visits per 1000 per year) (Table 2; eFigure 1 in Supplement 1).

### Beneficiary and Community Characteristics Associated With UC Utilization

In the multivariable negative binomial model for UC utilization in 2018 and 2019, there was an inverse association with age, with 9% lower adjusted UC utilization among individuals aged 75 to 84 years (IRR, 0.91 [95% CI, 0.91-0.92]) and 24% lower utilization (IRR, 0.76 [95% CI, 0.75-0.77]) among individuals aged 85 years or older compared with those aged 65 to 74 years (Table 3). There was a positive monotonic association between adjusted UC utilization and frailty score, with 56% higher utilization among beneficiaries in the fourth frailty quartile compared with those in the first quartile (IRR, 1.56 [95% CI, 1.54-1.58]). Females had 36% higher adjusted UC utilization compared with men (IRR, 1.36 [95% CI, 1.35-1.37]). There were also differences in UC utilization by race and ethnicity, with the lowest adjusted utilization among Black beneficiaries, who had 36% fewer UC visits per 1000 compared with White beneficiaries (adjusted IRR, 0.64 [95% CI, 0.63-0.65]). In addition, Medicaid-eligible beneficiaries had 43% lower adjusted UC utilization compared with those who were not Medicaid eligible (adjusted IRR, 0.57 [95% CI, 0.56-0.58]). Rural beneficiaries had 45% lower adjusted UC utilization compared with urban beneficiaries (IRR, 0.55 [95% CI, 0.54-0.55]). There was a monotonic inverse association between SDI quartile and UC visits, with 23% lower adjusted utilization (IRR, 0.77 [95% CI, 0.77-0.78]) among residents of communities in the fourth (most disadvantaged) quartile of SDI compared with those residing in the lowest quartile. Conversely, there was a positive monotonic association between the HRR-level quartile of physician supply and adjusted UC utilization.

**Table 3.** Association of Frailty and Demographic and Community Characteristics With Urgent Care Utilization in 2018 and 2019 Among Beneficiaries of Traditional Medicare

Characteristic	No. (%) of visits (n = 1 209 781)	Unadjusted No. of visits per 1000 beneficiary-years	Adjusted incidence rate ratio (95% CI)^a^
Age category, y			
65-74	687 735 (56.8)	209.5	1 [Reference]
75-84	378 700 (31.3)	214.5	0.91 (0.91-0.92)
≥85	143 346 (11.8)	178.2	0.76 (0.75-0.77)
Frailty index^b^			
First quartile (least frail)	208 075 (17.2)	159.4	1 [Reference]
Second quartile	281 955 (23.3)	216.2	1.30 (1.29-1.32)
Third quartile	314 643 (26.0)	243.3	1.49 (1.48-1.51)
Fourth quartile (most frail)	279 571 (23.1)	226.9	1.56 (1.54-1.58)
Missing	125 537 (10.4)	174.7	1.36 (1.34-1.38)
Sex			
Male	444 140 (36.7)	171.6	1 [Reference]
Female	765 641 (63.3)	234.5	1.36 (1.35-1.37)
Race and ethnicity^c^			
American Indian or Alaska Native	3376 (0.3)	122.4	0.79 (0.76-0.83)
Asian or Pacific Islander	23 284 (1.9)	140.2	0.73 (0.71-0.74)
Black	53 391 (4.4)	124.5	0.64 (0.63-0.65)
Hispanic	49 819 (4.1)	158.6	0.92 (0.90-0.93)
White	1 047 883 (86.6)	220.1	1 [Reference]
Other	8452 (0.7)	177.9	0.82 (0.79-0.84)
Unknown	23 576 (1.9)	218.8	1.04 (1.02-1.07)
Medicaid or eligibility			
Not Medicaid eligible	1 125 157 (93.0)	220.5	1 [Reference]
Medicaid eligible	84 624 (7.0)	112.7	0.57 (0.56-0.58)
Urban vs rural residence^d^			
Urban	1 043 284 (86.2)	232.9	1 [Reference]
Rural	165 285 (13.7)	121.6	0.55 (0.54-0.55)
Social Deprivation Index^e^			
First quartile (least disadvantaged)	423 941 (35.0)	258.2	1 [Reference]
Second quartile	348 818 (28.8)	215.8	0.91 (0.91-0.92)
Third quartile	251 936 (20.8)	173.8	0.83 (0.82-0.83)
Fourth quartile (most disadvantaged)	158 384 (13.1)	155.0	0.77 (0.77-0.78)
Missing	26 702 (2.2)	216.2	1.22 (1.19-1.25)
No. of physicians per 100 000 population^f^			
First quartile (fewest physicians)	261 897 (21.6)	180.7	1 [Reference]
Second quartile	286 778 (23.7)	199.9	1.07 (1.06-1.08)
Third quartile	309 351 (25.6)	214.5	1.14 (1.13-1.15)
Fourth quartile (most physicians)	350 288 (29.0)	233.1	1.17 (1.17-1.18)
Missing	1467 (0.1)	59.1	0.12 (0.11-0.14)

^a^ A negative binomial regression model was specified, with number of urgent care visits as the outcome and the natural log of year as the offset as well as beneficiary age, sex, Medicaid eligibility, frailty index, and race and ethnicity, as well as the following community characteristics associated with beneficiary residential zip code: urban vs rural location, Social Deprivation Index, and number of physicians per 100 000 population.

^b^ Frailty score was determined according to prior published claims-based methods.^20^

^c^ Race and ethnicity were defined using the Research Triangle Institute Variable. “Other” refers to all beneficiaries who cannot be assigned to 1 of the other 5 categories of race and ethnicity, whereas “Unknown” refers to individuals lacking any administrative data on race and ethnicity.

^d^ Beneficiaries residing in zip codes with a Rural-Urban Community Area code of 4 or greater were classified as having a rural residence and all others as urban.

^e^ Quartiles of community Social Deprivation Index based on beneficiary zip code, as an indicator of community-level socioeconomic status.

^f^ Each beneficiary was assigned to a Dartmouth Atlas Hospital Referral Region according to zip code. Physician supply for each Hospital Referral Region was obtained for the Dartmouth Atlas and quartiles of Hospital Referral Region–level physician supply were created.

### Trends in Clinician Specialty for UC Visits

**Figure 2.**
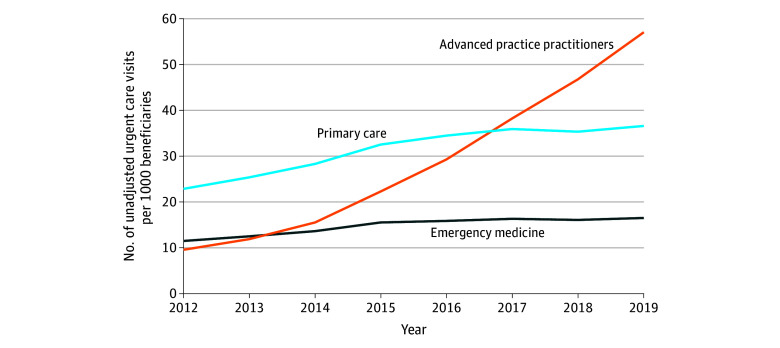
Urgent Care Utilization Among Medicare Beneficiaries by Year and Clinician Training and Specialty Unadjusted urgent care visits per 1000 traditional Medicare beneficiaries aged 65 years or older by year and clinician specialty. The 6 most common clinician specialties accounted for 97.3% of visits in all years and were aggregated into the following 3 categories: emergency medicine physicians, advanced practice practitioners (nurse practitioners and physician assistants), and physicians trained in primary care specialties (family practice, internal medicine, and general practice).

The following 6 clinician types accounted for 3 424 553 UC visits (97.4%) across all study years (eTable 1 in Supplement 1): family practice (n = 1 100 784 [31.3%]), physician assistant (n = 740 187 [21.1%]), emergency medicine (n = 672 732 [19.1%]), nurse practitioner (n = 576 199 [16.4%]), internal medicine (n = 249 686 [7.1%]), and general practice (n = 84 965 [2.4%]). We aggregated visits into the following 4 clinician categories: primary care specialties (family practice, internal medicine, and general practice), emergency medicine, APPs (physician assistants and nurse practitioners), and other physicians. In 2012, primary care physicians accounted for 127 345 of all 252 926 UC visits (50.3%), followed by emergency medicine physicians (64 054 of 252 926 [25.3%]) and APPs (53 220 of 252 926 [21.0%]), with all other clinicians accounting for 8307 of 252 926 visits (3.3%) (eFigure 2 in Supplement 1). APPs had the largest increase in UC visits per 1000 beneficiaries (6.7 [95% CI, 6.7-6.8] visits per 1000 per year; *P* < .001) (Figure 2; eTable 2 in Supplement 1) from 9.5 (95% CI, 9.5-9.6) visits per 1000 in 2012 to 57.0 (95% CI, 56.8-57.2) visits per 1000 in 2019, a 497% relative increase. The increases in UC visits per 1000 were modest for primary care physicians (1.8 [95% CI, 1.8-1.9] visits per 1000 beneficiaries per year; *P* < .001) as well as emergency medicine physicians (0.5 [95% CI, 0.5-0.6] visits per 1000 per year; *P* < .001). In 2019, APP visits accounted for 324 543 of 638 490 visits (50.8%), while all physician specialties all showed a decrease in their respective share of UC visits relative to 2012 (eFigure 2 in Supplement 1).

## Discussion

In this national cross-sectional study of UC visits among Medicare beneficiaries aged 65 years or older, we found that UC utilization more than doubled among this group over 8 years. These trends were not uniform; older beneficiaries, men, and members of racial and ethnic minority groups, as well as those who were eligible for Medicaid, saw smaller increases over time and lower adjusted UC utilization in recent years. Similarly, older adults in rural and socioeconomically disadvantaged communities had lower baseline rates of UC visits and lesser increases over time. The proportion of UC visits among older adults managed by physicians decreased, while visits managed by APPs increased, accounting for more than half of visits in 2019. Despite the marked increase in visits managed by APPs, areas with a lower supply of physicians had fewer increases in UC utilization. This trend could be due to several factors, including physician workforce shortages, financial obstacles in establishing new centers, and reduced economic viability of UC in areas of lower population density.^24^

The marked increase in UC utilization among older adults is consistent with the evolution of acute, unscheduled care delivery in recent years. Adults in the US are increasingly seeking episodic care at an ED, UC, or retail center over their primary care clinician when they are acutely ill.^1,25^ Unscheduled primary care access is low,^8,26,27^ and UC is thought to represent a convenient alternative to the ED with lower per-visit costs^4^ when a primary care clinician is not available.^28^ Although older adults have historically relied on the ED compared with younger populations,^5^ the present study suggests that older adults, too, are increasingly turning to UC centers. These trends are muted among those aged 85 years and older, which may be associated with medical complexity that exceeds the capabilities of most UC centers and/or with patient and care partner preferences.

However, the variable trends suggest that such growth has lagged among vulnerable patients and communities. This phenomenon is likely due to the disproportionate entry of UC centers into wealthy, urban communities^21^ and thus to limited geographic access, rather than differences in beneficiary preference for the site of care. Given that rural and economically disadvantaged communities also have a primary care shortage, office-based acute care options likely remain significantly limited, leaving the ED as the primary option during an acute illness. Given that EDs have become increasingly crowded in recent years^29^ and that ED crowding is associated with a range of adverse outcomes among older adults,^30^ the lack of alternative acute care options among vulnerable beneficiaries and communities may widen disparities in health care access and outcomes. A study in the Medicare population found that UC entry into a market was associated with no changes in mortality,^2^ but these data preceded the recent surge in ED crowding that accompanied the COVID-19 pandemic.^11^ From a health systems perspective, UC availability is thought to be associated with decreasing ED utilization among the commercially insured, in turn reducing revenue margins^31^ in emergency care, while the population served has become increasingly medically complex and publicly insured or uninsured.^32,33^ These concurrent trends threaten the financial viability of the ED safety net, which likely cross-subsidizes across reimbursement generosity to cover the fixed costs of emergency preparedness.^34^

The marked increase in UC visits managed by APPs is consistent with broader national trends,^35,36,37,38^ with APPs accounting for one-fourth of all visits across ambulatory and skilled nursing facility settings.^39^ Prior work suggests that APPs are not substituting for physicians on a one-for-one basis but disproportionately managing low-acuity conditions (eg, respiratory infections).^39^ Given that such conditions account for a large share of UC visits,^40^ the growth in APPs in this setting may seem unsurprising. However, given the idea that UC may substitute for the ED,^40^ access to advanced services is likely needed in many cases, particularly for older populations. In the present study, emergency medicine physicians managed 19.1% of UC visits, contrasting with the broader ambulatory care environment.^39^ Increasingly, there has been differentiation in types of UC centers, with some described as “advanced urgent care centers”^41^ staffed by emergency medicine physicians and nurses and offering a greater range of services compared with typical UC centers. However, time pressures and low reimbursement may limit the procedures done in such centers, even when the clinical capability exists. Given that a significant share of ED visits among older adults involves advanced imaging (eg, computed tomography scans),^42,43^ UC centers offering these services may be more effective at safely reducing ED referrals. The association of such centers with efficiency and quality of care warrants future study.

This study adds to a growing body of work highlighting the complex interplay between primary care, UC, and the ED. The increase in episodic visits to non–primary care settings is likely associated with barriers to accessing primary care, as well as patient preference for the convenience of on-demand, after-hours care.^44,45^ UC is thought to fill in the gaps in primary care access, as well as reduce the need for the ED. However, existing work suggests that UC entry has a limited association with ED use overall.^2,4^ Older age and socioeconomic disadvantage are shared risk factors for lower access to both primary care^44,46^ and UC, suggesting that the ED may continue to serve as the predominant acute care venue for vulnerable populations.^5^ Our study builds on this work by characterizing the magnitude of and variation in UC utilization among older adults, including the widening of disparities in access and the heterogeneity of clinician training that older adults may encounter during a UC visit.

### Limitations

This study has several limitations, including the examination of only traditional Medicare populations. Although our findings may not be generalizable to Medicare Advantage populations, prior work suggests that overall health care utilization is similar among those in Medicare Advantage and traditional Medicare in ambulatory and ED settings.^47^ Furthermore, we cannot ascertain why rural, socioeconomically disadvantaged populations have had fewer UC visits. Prior work suggests that inadequate access is a likely factor,^21^ and it is possible that patient preference or other unobserved characteristics may have played a role. Beneficiaries in rural areas use the ED^24^ as well as Federally Qualified Health Centers at higher rates compared with urban populations. Rural health clinics also provide acute, unscheduled care alongside preventive services. Future work examining how UC centers fit within the unique rural health care delivery landscape is needed.

In addition, when considering clinician specialty, it is possible that we may have underestimated the share of visits managed primarily by APPs due to indirect billing. Prior work among Medicare beneficiaries in ambulatory settings suggests that the share of physician visits that are “indirect billing” events, in which an APP managed care, was 6.9% in 2019, although there was variation by specialty.^39^

## Conclusions

In this cross-sectional analysis of older adults in the US, there was a marked increase in UC utilization, particularly in urban, high-income areas with a greater physician supply. Physicians in primary care specialties, emergency medicine physicians, and APPs accounted for most UC visits, with the greatest increase among APPs. These findings highlight the evolving patterns of acute care delivery for this growing population and the need for additional evidence on how these trends are associated with patient-centered outcomes and the efficiency of care.

## Notes

Supplement 1.
eAppendix.
**eFigure 1.** Trends in Urgent Care Visits per 1000 Eligible Beneficiaries by Beneficiary Sociodemographic and Community Characteristics **eTable 1.** Clinician Specialty/Training Associated With Urgent Care Professional Claims From 2012-2019 Among Beneficiaries of Fee-for-Service Medicare Ages 65 or Older **eFigure 2.** Percentage of Visits Attributed to Each Clinician Specialty by Year for Urgent Care Visits Among Traditional Medicare Beneficiaries Ages 65 or Older by Year From 2012-2019 **eTable 2.** Trends in Urgent Care Visits per 1000 Medicare Beneficiaries Ages 65 or Older by Clinician Specialty/Training

Supplement 2.
Data Sharing Statement
