# Supplementary material for: Trends in Urgent Care Utilization Among Medicare Beneficiaries From 2012 to 2019
Source: JAMA Netw Open. 2026 Jan 26;9(1):e2555345. doi: 10.1001/jamanetworkopen.2025.55345 (PMC12836136; doi:10.1001/jamanetworkopen.2025.55345)
Supplement: Supplement 1. — eAppendix. eFigure 1. Trends in Urgent Care Visits per 1000 Eligible Beneficiaries by Beneficiary Sociodemographic and Community Characteristics eTable 1. Clinician Specialty/Training Associated With Urgent Care Professional Claims From 2012-2019 Among Beneficiaries of Fee-for-Service Medicare Ages 65 or Older eFigure 2. Percentage of Visits Attributed to Each Clinician Specialty by Year for Urgent Care Visits Among Traditional Medicare Beneficiaries Ages 65 or Older by Year From 2012-2019 eTable 2. Trends in Urgent Care Visits per 1000 Medicare Beneficiaries Ages 65 or Older by Clinician Specialty/Training [file jamanetwopen-e2555345-s001.pdf]

Supplementary Online Content

Mantilla JJ, Burke RC, Orav EJ, et al. Trends in urgent care utilization among Medicare beneficiaries from 2012 to 2019. *JAMA Netw Open.* 2026;9(1):e2555345. doi:10.1001/jamanetworkopen.2025.55345

eAppendix.
eFigure 1. Trends in Urgent Care Visits per 1000 Eligible Beneficiaries by Beneficiary Sociodemographic and Community Characteristics
eTable 1. Clinician Specialty/Training Associated With Urgent Care Professional Claims From 2012-2019 Among Beneficiaries of Fee-for-Service Medicare Ages 65 or Older
eFigure 2. Percentage of Visits Attributed to Each Clinician Specialty by Year for Urgent Care Visits Among Traditional Medicare Beneficiaries Ages 65 or Older by Year From 2012-2019
eTable 2. Trends in Urgent Care Visits per 1000 Medicare Beneficiaries Ages 65 or Older by Clinician Specialty/Training

This supplementary material has been provided by the authors to give readers additional information about their work.

© 2026 Mantilla JJ et al. *JAMA Network Open.*

eAppendix.

The study included urgent care visits in the United States among a 20% sample of beneficiaries of traditional, fee-for-service Medicare enrolled in Parts A and B, ages 65 and older, from 2012 to 2019. UC visits were identified from the Medicare carrier professional claims using evaluation and management Healthcare Common Procedure Coding System/Current Procedural Terminology (HCPCS/CPT) codes (99201-99205 and 99211-99215) and place of service code of Urgent Care.

Beneficiary age, race, sex and Medicaid eligibility were determined yearly and assigned to each visit. Beneficiaries with one or more months of Medicaid eligibility in a calendar year were designated as Medicaid eligible for all visits in that year. We identified 26 beneficiary chronic conditions from the Chronic Conditions Warehouse (CCW) file using the prior calendar year. If the beneficiary was not eligible in the prior year, then the CCW variable was marked as missing. We used the same approach for beneficiary frailty score; we used prior years claims to calculate a frailty score and marked this variable as missing if the beneficiary was not enrolled in Medicare in the prior year.

Beneficiary community characteristics (rurality, Social Deprivation Index and physicians per 100,000 population) were determined based on beneficiary 9-digit zip code. Rurality was determined from the 2010 Rural-Urban Commuting Area codes dataset. A code of four or greater was considered rural. The Social Deprivation Index was from the 2015-2019 data release. The physicians per 100,000 population measure was from the Dartmouth Atlas Hospital and Physician Capacity 2011 dataset. The three data sources were linked to the beneficiary file based on the beneficiary's zip code of record for each year of eligibility.

When we created quartiles of variables (e.g.,beneficiary frailty score), the quartile cutpoints were tailored to the unit of analysis. For the trends analysis, the unit of analysis was beneficiary*year and thus the quartiles were calculated at the beneficiary*year level.

For the negative binomial models using the 2018-2019 data years, the quartiles for frailty and physicians per 100,000 population were calculated based on all unique beneficiaries in the dataset. If a beneficiary had two years of data, the first value was used. The negative binomial regression model had the number of urgent care visits as the outcome and the natural log of the number of follow-up years as the offset, as well beneficiary age, sex, Medicaid eligibility, frailty index, race as well as the following community characteristics associated with beneficiary residential zip code: urban vs. rural location, social deprivation index and physicians per 100,000 population.

© 2026 Mantilla JJ et al. *JAMA Network Open.*

eFigure 1. Trends in Urgent Care Visits per 1000 Eligible Beneficiaries by Beneficiary Sociodemographic and Community Characteristics

1A. Urgent Care Visits per 1,000 by Beneficiary Age Category

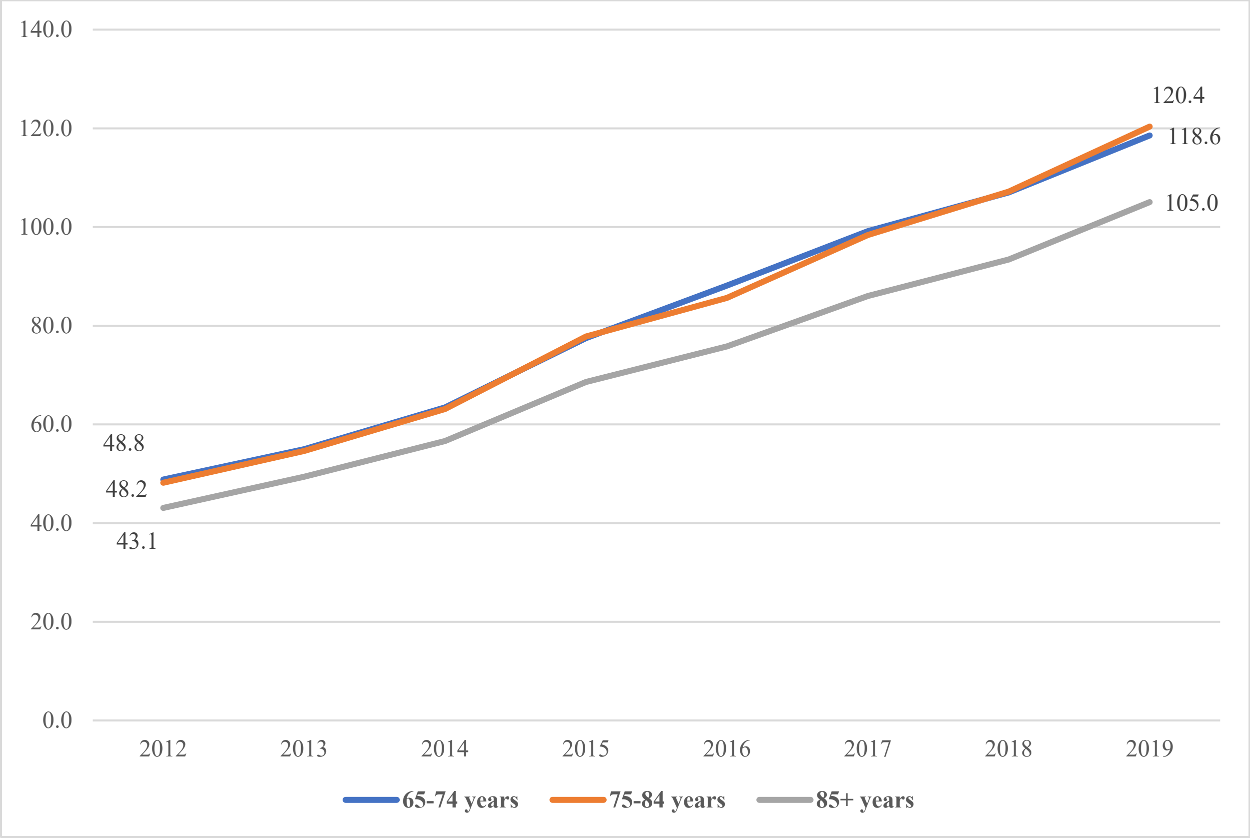

© 2026 Mantilla JJ et al. *JAMA Network Open.*

eFigure 1. Trends in Urgent Care Visits per 1,000 Eligible Beneficiaries by Beneficiary Sociodemographic and Community Characteristics

1B. Trends in Urgent Care Visits from 2012-2019 Among Medicare Beneficiaries Overall and Stratified by Beneficiary Frailty[a]

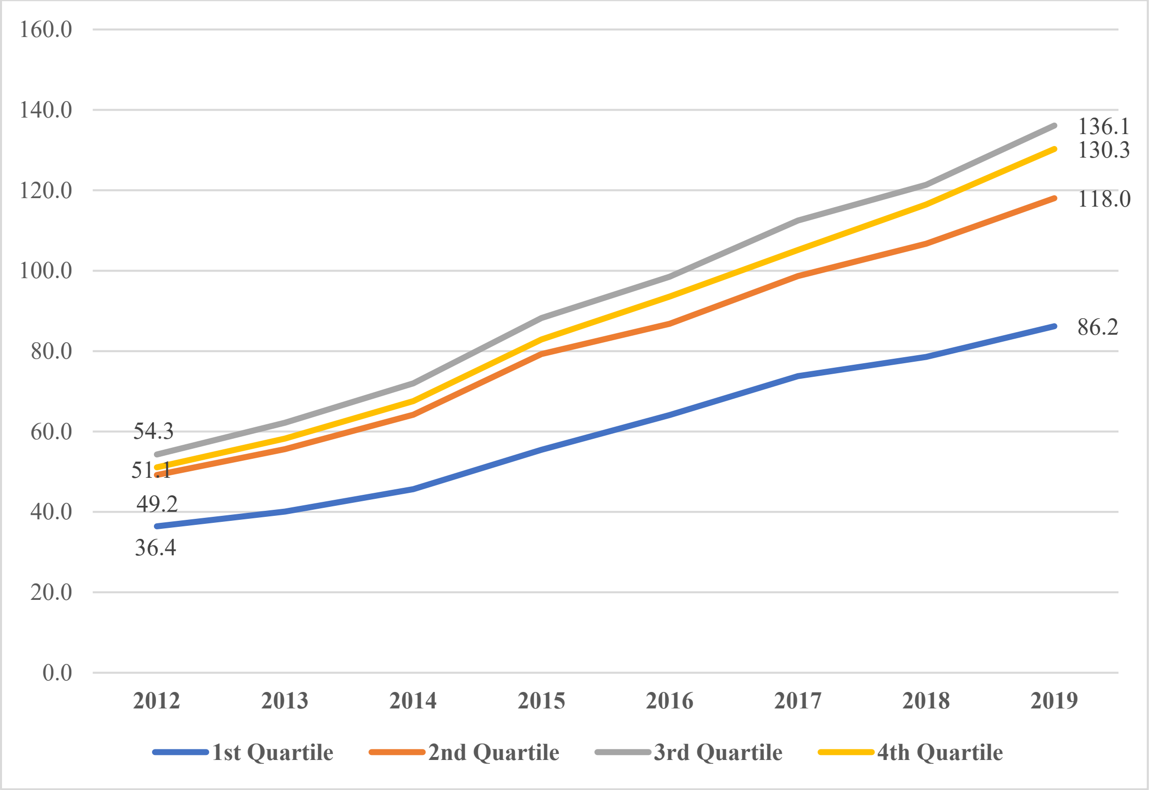

[a]Unadjusted urgent care visits per 1000 traditional Medicare Beneficiaries ages 65 and older by year and frailty quartile. Beneficiary frailty was calculated using previously described methods (Kim DH et al., J Gerontol A Biol Sci Med Sci. 2018;73(7):980-7.) and each beneficiary was assigned a quartile of frailty score.

© 2026 Mantilla JJ et al. *JAMA Network Open.*

eFigure 1. Trends in Urgent Care Visits per 1,000 Eligible Beneficiaries by Beneficiary Sociodemographic and Community Characteristics

1C. Urgent Care Visits per 1,000 by Year and Beneficiary Sex

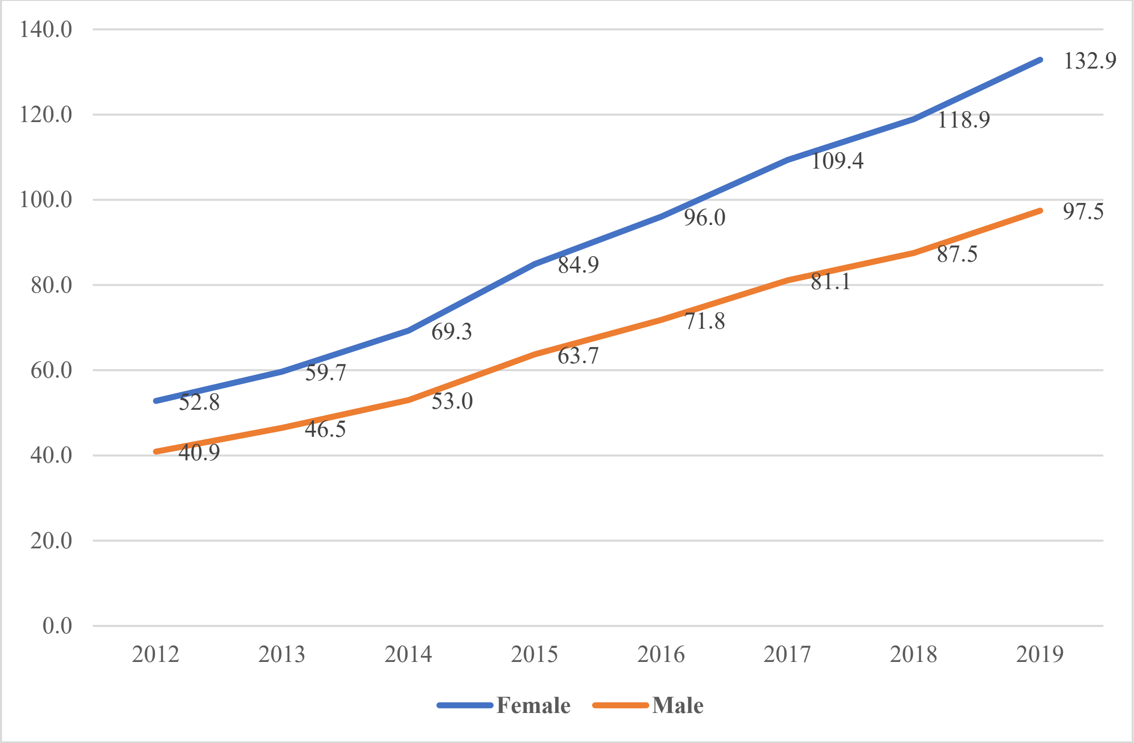

© 2026 Mantilla JJ et al. *JAMA Network Open.*

eFigure 1. Trends in Urgent Care Visits per 1,000 Eligible Beneficiaries by Beneficiary Sociodemographic and Community Characteristics

1D. Urgent Care Visits per 1,000 by Year and Beneficiary Race/Ethnicity[a]

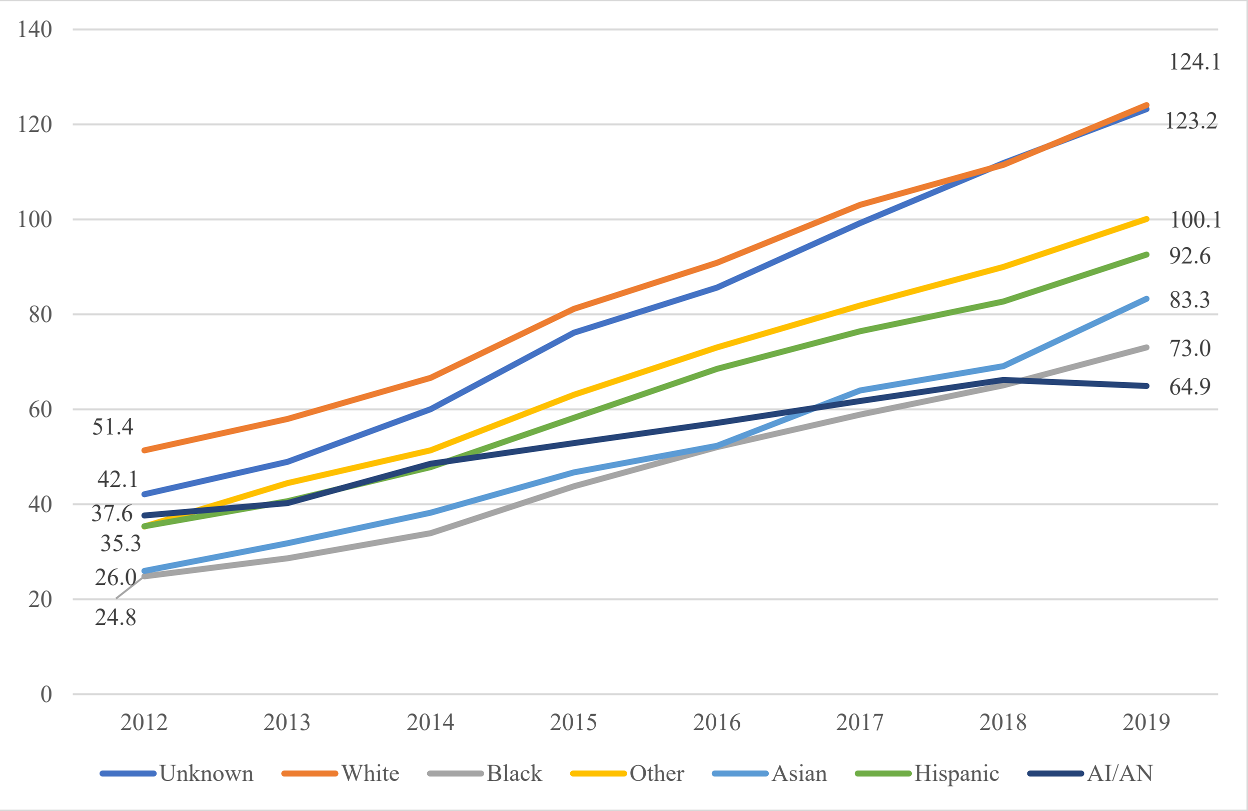

[a]Race/ethnicity was defined using the Research Triangle Institute Variable, "Other" refers to all beneficiaries who cannot be assigned to one of the other five categories of race/ethnicity, whereas "Unknown" refers to individuals lacking any administrative data on race/ethnicity. The abbreviation AI/AN refers American Indian/Alaska Native.

© 2026 Mantilla JJ et al. *JAMA Network Open.*

eFigure 1. Trends in Urgent Care Visits per 1,000 Eligible Beneficiaries by Beneficiary Sociodemographic and Community Characteristics

1E. Urgent Care Visits per 1,000 by Year and Medicaid Eligibility[a]

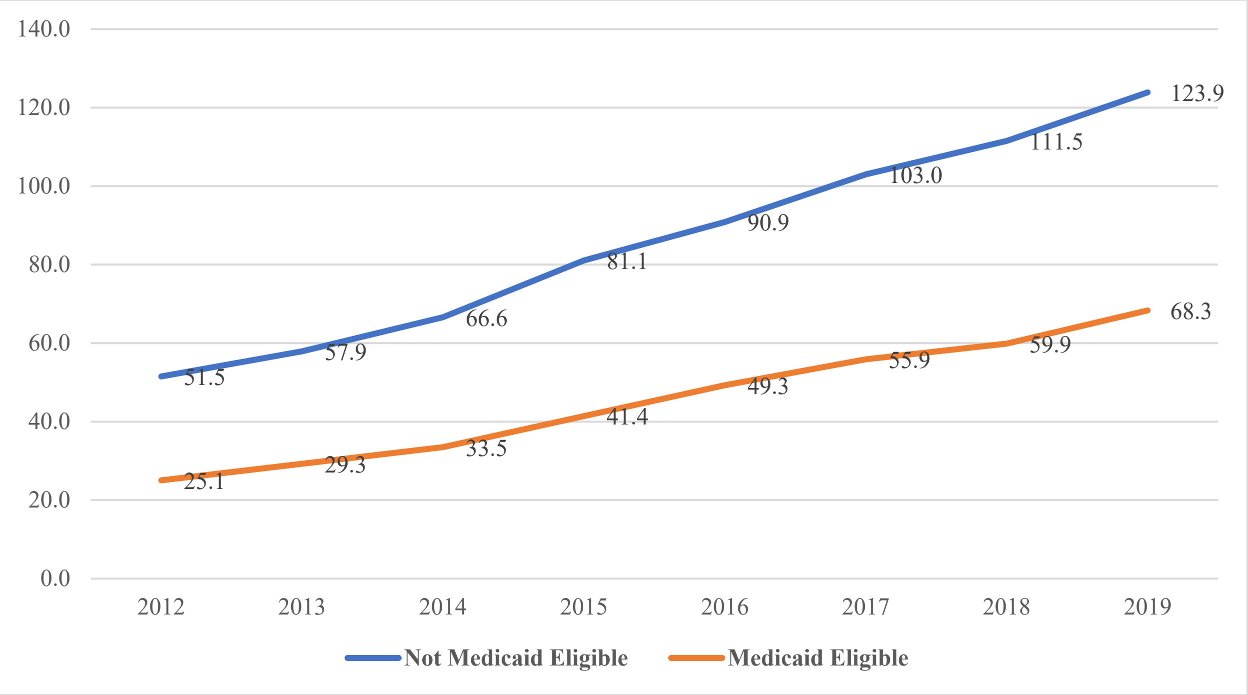

[a] Beneficiaries with at least 1 month of Medicaid eligibility in the year of the urgent care visit.

© 2026 Mantilla JJ et al. *JAMA Network Open.*

eFigure 1. Trends in Urgent Care Visits per 1,000 Eligible Beneficiaries by Beneficiary Sociodemographic and Community Characteristics

1F. Urgent Care Visits per 1,000 by Year and Beneficiary Zip-Code Quartile of Social Deprivation Index[a]

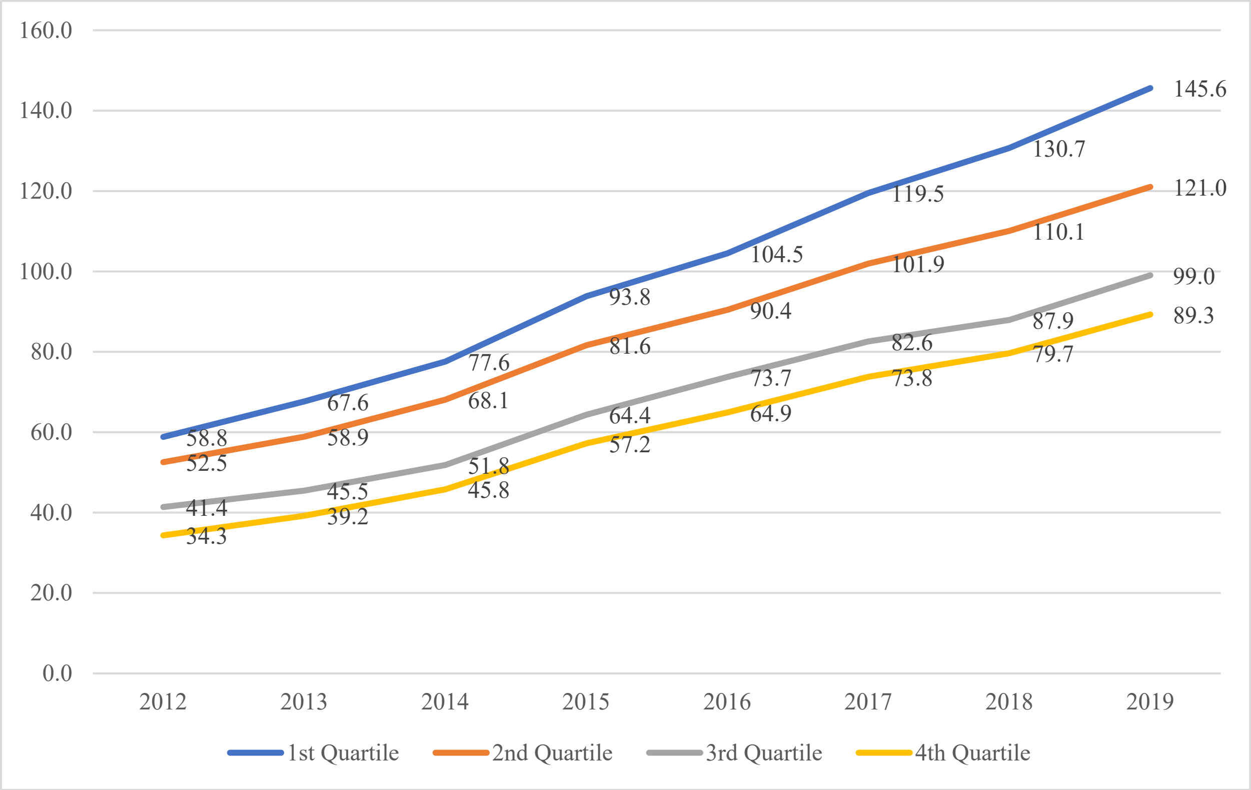

[a] Graham Social Deprivation Index determined at the beneficiary 9-digit zip code level. Butler DC, Petterson S, Phillips RL, Bazemore AW. Measures of Social Deprivation That Predict Health Care Access and Need within a Rational Area of Primary Care Service Delivery. Health Services Research. 2013;48(2 Pt 1):539-559. doi:10.1111/j.1475-6773.2012.01449.

© 2026 Mantilla JJ et al. *JAMA Network Open.*

eFigure 1. Trends in Urgent Care Visits per 1,000 Eligible Beneficiaries by Beneficiary Sociodemographic and Community Characteristics

1G. Urgent Care Visits per 1,000 by Year and Beneficiary Urban vs. Rural Residence[a]

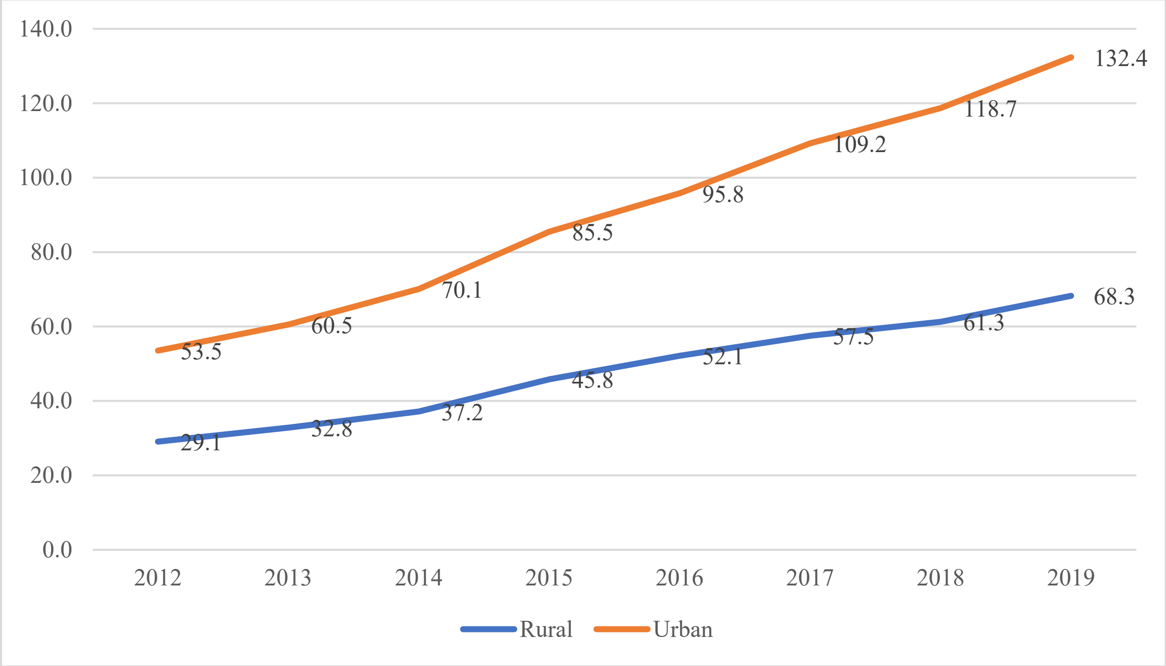

[a] Beneficiary urban vs. rural residence defined by Rural-Urban Commuting Area (RUCA) codes with RUCA codes 0-3 as urban and ≥ 4 as rural.

© 2026 Mantilla JJ et al. *JAMA Network Open.*

eFigure 1. Trends in Urgent Care Visits per 1,000 Eligible Beneficiaries by Beneficiary Sociodemographic and Community Characteristics

1H. Urgent Care Visits per 1,000 by Year and Physician Supply[a]

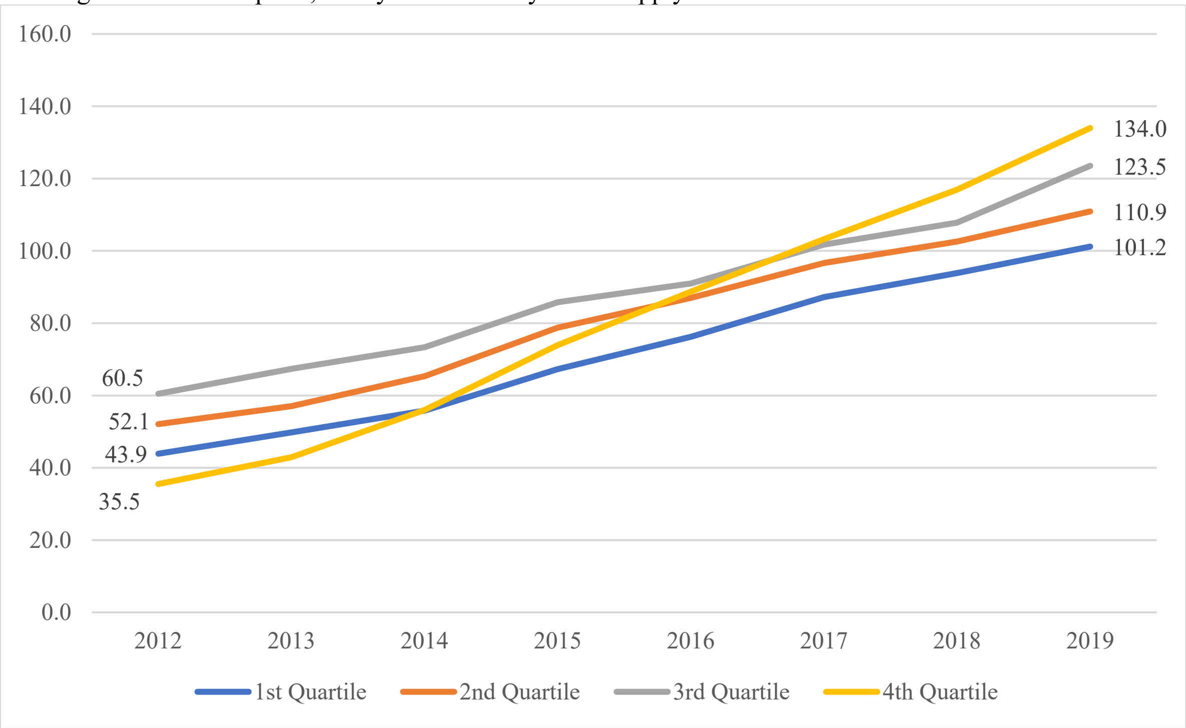

[a]Quartile of physicians per 100,000 population for the Hospital Referral Region in which the beneficiary resided in the year of the urgent care visit

© 2026 Mantilla JJ et al. *JAMA Network Open.*

eTable 1. Clinician Specialty/Training Associated With Urgent Care Professional Claims From 2012-2019 Among Beneficiaries of Fee-for-Service Medicare Ages 65 or Older

Clinician Specialty Name and Code	Visits (N)	Percent
**Family Practice - 8**	1,100,784	31.3%
**Physician assistant - 97**	740,187	21.1%
**Emergency medicine - 93**	672,732	19.1%
**Nurse practitioner - 50**	576,199	16.4%
**Internal medicine - 11**	249,686	7.1%
**General practice - 1**	84,965	2.4%
**Pediatric medicine - 37**	20,171	0.6%
**General surgery - 2**	13,204	0.4%
**Osteopathic manipulative therapy - 12**	6,548	0.2%
**Preventive medicine - 84**	5,256	0.2%
**Orthopedic surgery - 20**	4,604	0.1%
**Obstetrics/gynecology - 16**	4,499	0.1%
**Geriatric medicine - 38**	3,020	0.09%
**Anesthesiology - 5**	2,356	0.07%
**Otolaryngology - 4**	2,135	0.06%
**Unknown physician specialty - 99**	2,127	0.06%
**Cardiology - 6**	2,078	0.06%
**Pulmonary disease - 29**	1,976	0.06%
**Ophthalmology - 18**	1,718	0.05%
**Infectious disease - 44**	1,694	0.05%
**Physical medicine and rehabilitation - 25**	1,400	0.04%
**Dermatology - 7**	1,255	0.04%
**Critical care (intensivists) - 81**	1,233	0.04%
**Interventional Pain Management (IPM) - 9**	1,208	0.03%
**Nuclear medicine - 36**	1,135	0.03%
**Pathology - 22**	1,134	0.03%
**Neurology - 13**	1,130	0.03%
**Endocrinology - 46**	1,067	0.03%
**Nephrology - 39**	976	0.03%
**Rheumatology - 66**	968	0.03%
**Sports Medicine - 23**	911	0.03%
**Diagnostic radiology - 30**	896	0.03%
**Hospitalist - C6**	753	0.02%
**Hematology/oncology - 83**	649	0.02%
**Gastroenterology - 10**	645	0.02%
**Certified clinical nurse specialist - 89**	582	0.02%

© 2026 Mantilla JJ et al. *JAMA Network Open.*

**Neurosurgery - 14**	464	0.01%
**Urology - 34**	462	0.01%
**Psychiatry - 26**	405	0.01%
**Allergy/immunology - 3**	373	0.01%
**Colorectal surgery - 28**	349	0.01%
**Addiction medicine - 79**	315	0.01%
**Plastic and reconstructive surgery - 24**	264	0.01%
**Hospice and Palliative Care - 17**	261	0.01%
**Surgical oncology - 91**	216	0.01%
**Medical oncology - 90**	214	0.01%
**Podiatry - 48**	199	0.01%
**Single or Multispecialty clinic or group practice (PA Group)- 70**	196	0.01%
**Sleep medicine - C0**	193	0.01%
**Multispecialty clinic or group practice - 70**	190	0.01%
**Pain Management - 72**	191	0.01%
**Thoracic surgery - 33**	147	0.00%
**Hand surgery - 40**	146	0.00%
**Certified nurse midwife - 42**	146	0.00%
**Vascular surgery - 77**	132	0.00%
**Interventional radiology - 94**	92	0.00%
**Radiation oncology - 92**	44	0.00%
**Cardiac surgery - 78**	40	0.00%
**Chiropractic - 35**	17	0.00%
**Mass Immunization Roster Biller - 73**	17	0.00%
**Interventional cardiology - C3**	12	0.00%
**Registered Dietician/Nutrition Professional - 71**	<11	
**Independent Diagnostic Testing Facility (IDTF) - 47**	<11	
**Optometrist - 41**	<11	
**Undersea and Hyperbaric Medicine - D4**	<11	
**Gynecologist/oncologist - 98**	<11	
**Physical therapist (private practice) - 65**	<11	
**Unknown supplier/provider specialty - 88**	<11	
**Cardiac Electrophysiology -21**	<11	
**Clinical psychologist - 68**	<11	
**Medical Toxicology - C8**	<11	

© 2026 Mantilla JJ et al. *JAMA Network Open.*

eFigure 2. Percentage of Visits Attributed to Each Clinician Specialty by Year for Urgent Care Visits Among Traditional Medicare Beneficiaries Ages 65 or Older by Year From 2012-2019

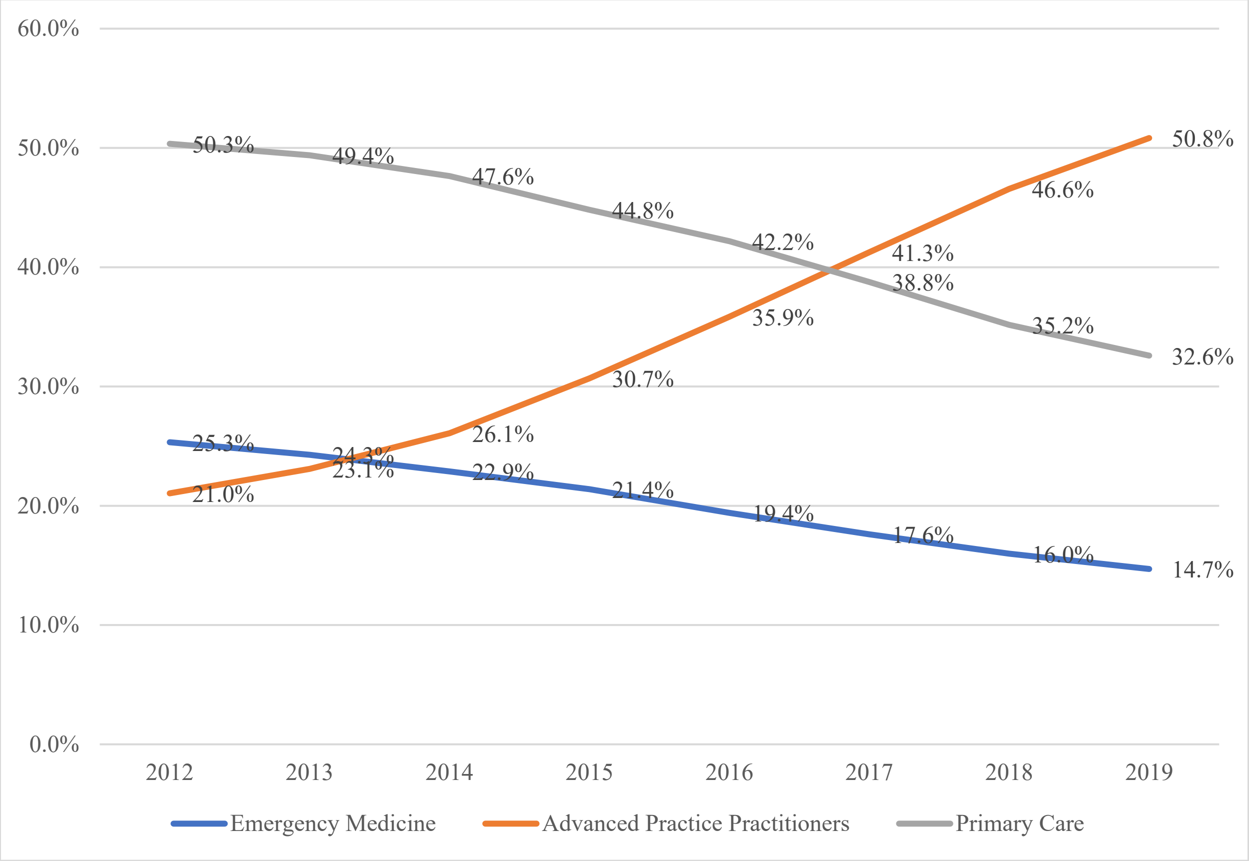

*Raw percentage of urgent care visits by year among a random 20% sample of traditional Medicare beneficiaries ages 65 and older by clinician specialty and training on the urgent care visit professional claim for evaluation and management services. The six most common clinician specialties accounted for 97.3% of visits in all years and were aggregated into the following three categories: emergency medicine physicians, advanced practice practitioners (APPs, consisting of nurse practitioners and physician assistants) and physicians trained in primary care specialties (family practice, internal medicine and general practice).

© 2026 Mantilla JJ et al. *JAMA Network Open.*

eTable 2. Trends in Urgent Care Visits per 1000 Medicare Beneficiaries Ages 65 or Older by Clinician Specialty

	Visits per 1000 Beneficiaries 2012[a]	Visits per 1000 Beneficiaries 2019[a]	Time trend (change in visits per 1000 beneficiaries per year) with 95% CI[b]
**Primary care physician specialties** (family medicine, internal medicine, general practice)	22.8	36.6	+1.83 (1.77-1.89)
**Emergency Medicine**	11.5	16.5	+0.53 (0.48-0.59)
**Advanced Practice Practitioners** (Nurse Practitioners, Physician Assistants)	9.5	57.0	+6.75 (6.69-6.80)

[a]Raw number of urgent care visits per 1000 traditional Medicare beneficiaries in the respective year. [b]Trends over time in urgent care visits per beneficiary as the outcome and year, clinician specialty (primary care, emergency medicine, advanced practice practitioners, and all other clinicians) as well an interaction between clinician specialty and years as the predictor.

© 2026 Mantilla JJ et al. *JAMA Network Open.*
