# Supplementary material for: Trends in Urgent Care Utilization Among Medicare Beneficiaries From 2012 to 2019
Source: JAMA Netw Open. 2026 Jan 26;9(1):e2555345. doi: 10.1001/jamanetworkopen.2025.55345 (PMC12836136; doi:10.1001/jamanetworkopen.2025.55345)
Supplement: Supplement 2. — Data Sharing Statement [file jamanetwopen-e2555345-s002.pdf]

Data Sharing Statement

Mantilla. Trends in Urgent Care Utilization Among Medicare Beneficiaries From 2012 to 2019. *JAMA Netw Open*. Published January 26, 2026. doi:10.1001/jamanetworkopen.2025.55345

Data
Data available: No
